# Revision of MELD to Include Serum Albumin Improves Prediction of Mortality on the Liver Transplant Waiting List

**DOI:** 10.1371/journal.pone.0051926

**Published:** 2013-01-18

**Authors:** Robert P. Myers, Abdel Aziz M. Shaheen, Peter Faris, Alexander I. Aspinall, Kelly W. Burak

**Affiliations:** Liver Unit, Division of Gastroenterology and Hepatology, Department of Medicine, University of Calgary, Calgary, Alberta, Canada; University of Colorado, United States of America

## Abstract

**Background:**

Allocation of donor livers for transplantation in most regions is based on the Model for End-Stage Liver Disease (MELD) or MELD-sodium (MELDNa). Our objective was to assess revisions to MELD and MELDNa that include serum albumin for predicting waiting list mortality.

**Methods:**

Adults registered for liver transplantation in the United States (2002–2007) were identified from the United Network for Organ Sharing (UNOS) database. Cox regression was used to determine the association between serum albumin and 3-month mortality, and to derive revised MELD and MELDNa scores incorporating albumin (‘MELD-albumin’ and ‘5-variable MELD [5vMELD]’).

**Results:**

Among 40,393 patients, 9% died and 24% underwent transplantation within 3 months of listing. For serum albumin concentrations between 1.0 and 4.0 g/dL, a linear, inverse relationship was observed between albumin and 3-month mortality (adjusted hazard ratio per 1 g/dL reduction in albumin: 1.44; 95% CI 1.35–1.54). The c-statistics for 3-month mortality of MELD-albumin and MELD were 0.913 and 0.896, respectively (*P*<0.001); 5vMELD was superior to MELDNa (c-statistics 0.922 vs. 0.912, *P*<0.001). The potential benefit of 5vMELD was greatest in patients with low MELD (<15). Among low MELD patients who died, 27% would have gained ≥10 points with 5vMELD over MELD versus only 4–7% among low MELD survivors and high MELD (≥15) candidates (*P*<0.0005).

**Conclusion:**

Modification of MELD and MELDNa to include serum albumin is associated with improved prediction of waiting list mortality. If validated and shown to be associated with reduced mortality, adoption of 5vMELD as the basis for liver allograft allocation may improve outcomes on the liver transplant waiting list.

## Introduction

In February 2002, the Model for End-Stage Liver Disease (MELD) score replaced the Child-Turcotte-Pugh (CTP) score for the prioritization of potential liver transplant recipients in the United States. Since then, numerous other regions have adopted a MELD-based allocation policy. Whereas MELD includes only objective laboratory parameters (the international normalized ratio [INR] of the prothrombin time, and serum bilirubin and creatinine), the CTP score includes both objective (INR, bilirubin, and albumin) and subjective components (ascites and encephalopathy). Although MELD was developed to predict survival following elective transjugular intrahepatic portosystemic shunt (TIPS) insertion, its primary use currently is the prediction of short-term mortality in cirrhotic patients on the liver transplant waiting list [1], [2], [3], [4], [5]. In the original TIPS cohort, a negative association was observed between the serum albumin concentration and 3-month mortality in a univariate analysis that was not significant in a multivariate model, perhaps due to the small sample size of the study [1]. As such, albumin was excluded from the final MELD model; however, it is a component of the Pediatric End-Stage Liver Disease (PELD) score used to prioritize pediatric liver transplant candidates [6].

Since implementation of a MELD-based allocation policy in the United States, reductions in the number of patients listed for liver transplantation [4], deaths on the waiting list [7], and median wait times have been reported. Despite these benefits, limitations of MELD have been recognized and attempts are ongoing to refine it [8]. Proposed modifications include reweighting the model's coefficients [9], altering its laboratory components [10], [11], [12], and the addition of new variables [13], [14]. For example, Kim *et al.* reported that the addition of the serum sodium concentration to generate the MELDNa score was more accurate than MELD for predicting short-term mortality on the waiting list [13]. The authors estimated that use of MELDNa might have prevented 7% of deaths that occurred within 90 days of listing. In a subsequent study using the same database, we reported a significant negative impact of hypoalbuminemia on waiting list mortality after adjustment for MELD, serum sodium, and other important covariates [10].

In light of the limitations of MELD and the potential benefits of incorporating serum albumin, we used a nationwide database to evaluate the association between serum albumin and mortality on the liver transplant waiting list. Our primary objective was to determine if the addition of albumin to MELD and MELDNa could improve prediction of short-term mortality. We also evaluated the interaction between the serum albumin concentration and MELD, to determine if the association between albumin concentration and mortality is consistent across the entire spectrum of MELD scores.

## Methods

### Data Source and Study Population

**Figure 1:**
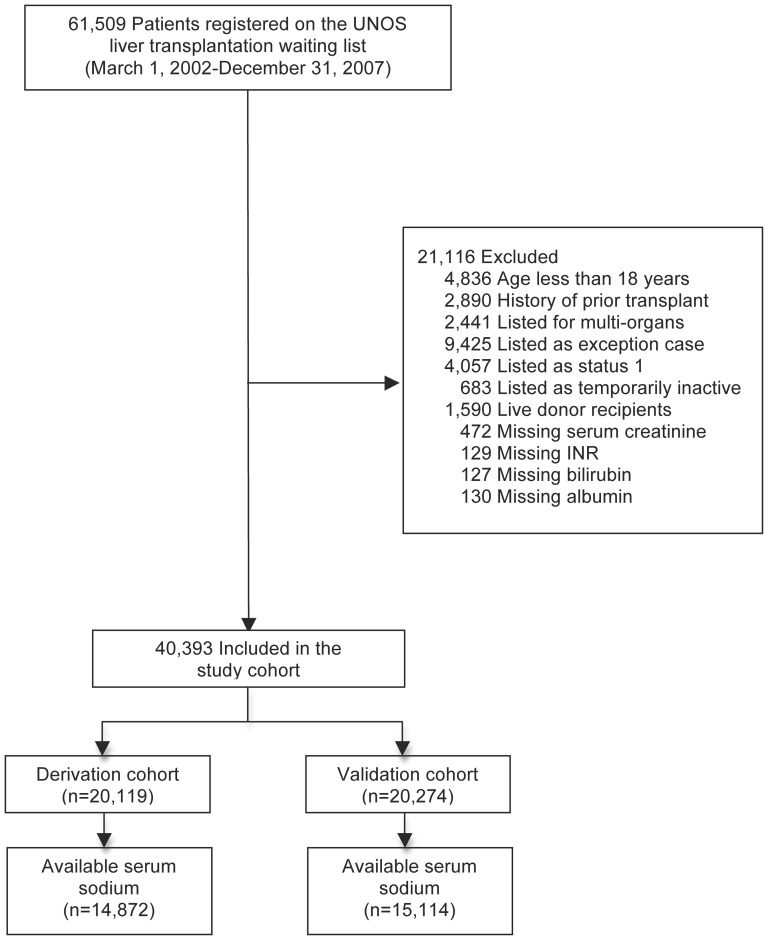
Flow diagram of patients registered on the UNOS liver transplant waiting list in the United States between March 1, 2002 and December 31, 2007. Only patients with available serum sodium concentrations were included in statistical comparisons between models.

The United Network for Organ Sharing (UNOS) Standard Transplant Analysis and Research (STAR) database was used to identify patients registered on the liver transplant waiting list in the United States between March 1, 2002 and December 31, 2007. Adults 18 years and over whom were listed for their first liver transplantation were included. Patients listed for multiple organs and live donor liver recipients were excluded (Figure 1). Given the different criteria for organ allocation, we also excluded patients listed as status 1 (e.g. with acute liver failure), temporarily inactive, and exception cases (e.g. with hepatocellular carcinoma, hepatopulmonary syndrome, etc.). Finally, patients with missing laboratory data, including serum albumin and tests necessary for calculation of MELD, were excluded.

### Variables of Interest

Our primary exposure variable was the serum albumin concentration (measured in g/dL). Additional predictor variables included age, gender, race (categorized as white, black, Hispanic, and other), hepatic diagnosis (categorized as hepatitis C, alcoholic liver disease, cholestatic liver disease, and other), body weight (as a reflection of organ size matching), blood group, UNOS region, and the INR, bilirubin, sodium, and creatinine at the time of registration on the waiting list. The MELD and MELDNa scores were calculated according to previously published formulas (see the Appendix).

### Statistical Analyses

**Figure 2:**
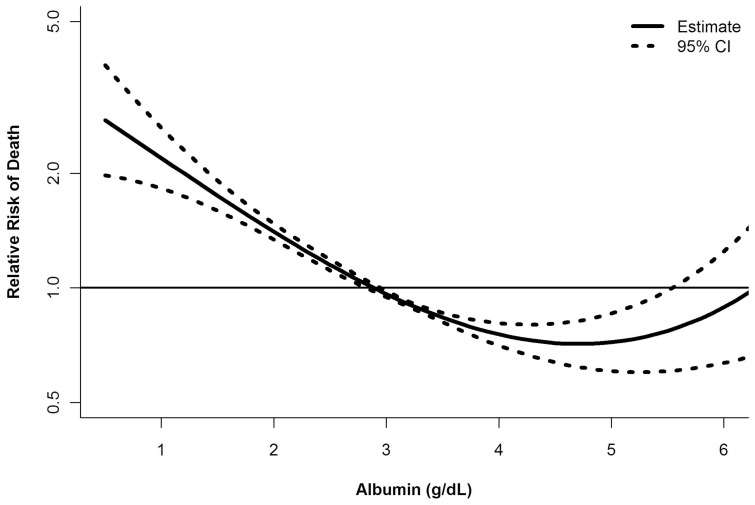
Serum albumin concentration and the risk of death within 3 months of waiting list registration after adjustment for the MELD score. Dotted lines represent 95% confidence intervals.

Comparisons between groups were made using Fisher's exact and chi^2^ tests for categorical variables and Mann-Whitney and Kruskal-Wallis rank tests for continuous variables. The association between serum albumin and mortality on the liver transplant waiting list was assessed using Kaplan-Meier analysis and Cox regression. Our primary outcome was all-cause mortality within 90 days of waiting list registration. Patients were censored at transplantation, the end of follow-up (December 31, 2008), or withdrawal from the list, whichever came first. Individuals removed because they were ‘too sick to transplant’ were counted as deaths (n = 2,596). Supplementary analyses that censored these patients on the day of removal (i.e. considered them survivors) yielded nearly identical results (data not shown). As secondary outcomes, we assessed transplantation at 90 days and mortality at one year and during the entire follow-up period. Multivariate models adjusted for age, gender, race, diagnosis, body weight, blood group, UNOS region, MELD score, and serum sodium at registration. To determine whether serum albumin had a non-linear effect on the risk of mortality, we examined generalized additive models with smoothing splines. The resultant smooth curves enabled examination of the relationship between mortality and MELD across several strata of serum albumin [15]. We present the curve showing the relationship between serum albumin and the risk of mortality after adjusting for MELD (Figure 2).

#### Model Derivation

Our primary objective was to develop predictive models for 3-month mortality on the transplant waiting list akin to MELD and MELDNa that include serum albumin. To do so, we randomly divided the study cohort into derivation and validation groups. The predictive models, referred to as ‘MELD-albumin’ and ‘5-variable MELD (5vMELD)’ were derived using Cox proportional hazards models with 3-month mortality as the dependent variable and MELD (or MELDNa), serum albumin, and an interaction term as independent variables. A supplementary analysis that used competing risks regression and considered transplantation within 3 months as a competing event yielded very similar results (data not shown). Since we were interested in the impact of hypoalbuminemia, 30 patients (0.15%) with hyperalbuminemia (serum albumin >5.0 g/dL) were excluded from model development. Including these patients in model derivation did not affect the results (data not shown). To remain consistent with MELD and MELDNa, the final models were rounded to the nearest integer and constrained between 6 and 40.

#### Model Performance

Discrimination of the novel models was determined in the validation cohort and compared with MELD and MELDNa. Discrimination, which refers to a model's ability to correctly distinguish between two outcomes (i.e. death or survival on the waiting list), was assessed using the concordance statistic (c-statistic) modified for survival data. The c-statistic from the Cox model is analogous to the area under a receiver operating characteristic curve estimated for logistic models [16], [17]. *P*-values for comparisons of c-statistics were calculated using the group jackknife method [18]. For these analyses, the cohort was limited to patients with available serum sodium (n = 15,114 [75%]) to permit valid comparisons of the four models. Due to limitations in the availability of serum sodium during the earlier years, 72% of these patients (n = 10,817) were listed during the latter half of the study (2005 to 2007). Supplementary analyses including the entire validation cohort (n = 20,274) revealed similar results (data not shown).

#### Reclassification of Mortality Risk

Because differences in c-statistics are difficult to conceptualize clinically, we generated risk reclassification tables [19], [20], [21], [22]. Reclassification of the risk of mortality at 90 days was evaluated by comparing predicted risk estimates from a Cox model for MELD (the current standard for organ allocation) with those of models for MELD-albumin, 5vMELD, and MELDNa considering the number of deaths actually observed within strata. To conduct these analyses, 90-day mortality estimates were grouped into the following risk strata: 0 to <5%, 5% to <10%, 10% to <20%, and ≥20%. For each model, we calculated reclassification calibration statistics, which are analogous to the Hosmer-Lemeshow goodness-of-fit statistic, but applied to reclassified categories [21]. We also evaluated reclassification rates separately in individuals who died compared with survivors to reflect the fact that not all reclassifications are beneficial for risk prediction. For example, in individuals who die and are reclassified by a new model into a higher risk category, reclassification is considered beneficial. On the contrary, any downward movement in category among decedents implies worse reclassification. The opposite is true for survivors. The net reclassification improvement (NRI) was therefore calculated by summing the reclassification improvements for decedents and survivors [21], with *P*-values and 95% confidence intervals (CI) determined using asymptotic tests.

#### Potential Survival Benefit of 5vMELD in Patients with Low MELD Scores

We hypothesized that a 5vMELD-based allocation system would be most beneficial among patients with low MELD scores (<15). Because transplantation of these patients under MELD is less likely, they may incur a significant risk of death in settings with long waiting times. To address this hypothesis, we calculated the difference between 5vMELD and MELD in the validation cohort. Patients who would gain ≥10 points with 5vMELD were considered reflective of those who would achieve a meaningful increase in the likelihood of transplantation under a 5vMELD-based system. We compared the median difference in 5vMELD versus MELD and the proportion with a ≥10-point difference within four strata defined by listing MELD score (low vs. high) and 3-month mortality. A secondary analysis examined a gain of ≥5 points.

All analyses were conducted using SAS (v9.2; SAS Institute; Carey, NC) and Stata/SE (v11.0; StataCorp LP; College Station, TX). Two-sided *P*-values less than 0.05 were considered statistically significant.

## Results

### Patient Characteristics

A total of 61,509 patients were registered on the UNOS liver transplant waiting list between March 1, 2002 and December 31, 2007. Of these, 40,393 patients satisfied the eligibility criteria (Figure 1); their characteristics are outlined in Table 1. There were no significant differences between patients in the derivation and validation cohorts (data not shown). At registration, the median MELD score was 15 (interquartile range [IQR] 11–20) and the median serum albumin concentration was 3.0 g/dL (IQR 2.5–3.4 g/dL; range 0.5–8.4 g/dL). Overall, 76% of patients were hypoalbuminemic (serum albumin <3.5 g/dL), and in 6% of patients, the serum albumin concentration was <2.0 g/dL. Only 62 patients (0.15%) were hyperalbuminemic (serum albumin >5.0 g/dL). Compared with patients with normal serum albumin concentrations, hypoalbuminemic patients tended to have higher MELD scores, including greater serum bilirubin and INR, and lower serum sodium concentrations (Table 1). Hyperalbuminemic patients had worse hepatic and renal dysfunction, including greater MELD scores and a higher proportion requiring dialysis (16% vs. 4% in the other groups; *P* = 0.0001). Serum albumin was inversely correlated with MELD (Spearman's rho = −0.41) and MELDNa (rho = −0.44), but positively correlated with serum sodium (rho = 0.30; all *P*<0.0001).

**Table 1:** Characteristics of Registrants on the Liver Transplant Waiting List.

Variable	Total Cohort (n = 40,393)	Hypoalbuminemia (Albumin <3.5 g/dL) (n = 30,656)	Normal Albumin (Albumin 3.5–5.0 g/dL) (n = 9,675)	Hyperalbuminemia (Albumin >5.0 g/dL) (n = 62)	*P*-value
**Female sex**	36%	35%	37%	40%	<0.001
**Age, ** ***yrs***	53 (47–59)	53 (47–59)	53 (47–59)	50 (44–57)	0.005
**Race or ethnic group** *					
White	74%	73%	76%	74%	<0.001
Black	7%	8%	6%	3%	
Hispanic	14%	15%	12%	16%	
Other	5%	4%	5%	6%	
**Diagnosis**					
Hepatitis C	40%	43%	33%	31%	<0.001
Alcohol	17%	16%	19%	24%	
Cholestasis	9%	9%	11%	2%	
NAFLD/cryptogenic	12%	12%	13%	15%	
Metabolic ¥	2%	2%	2%	2%	
Other	20%	19%	22%	27%	
**Body weight, ** ***kg***	83 (70–96)	84 (71–97)	80 (68–93)	73 (65–97)	<0.001
**MELD score**	15 (11–20)	16 (13–21)	12 (9–16)	26 (17–35)	<0.001
**Bilirubin (total), ** ***mg/dL***	2.5 (1.4–4.9)	2.8 (1.7–5.4)	1.5 (0.9–2.9)	3.4 (1.9–14.1)	<0.001
**Creatinine, ** ***mg/dL***	1.0 (0.8–1.3)	1.0 (1.0–1.3)	1.0 (1.0–1.2)	1.9 (1.0–3.4)	<0.001
**INR**	1.4 (1.2–1.7)	1.5 (1.3–1.8)	1.2 (1.1–1.4)	1.7 (1.3–2.7)	<0.001
**Sodium, ** ***mmol/L*** †	137 (134–140)	136 (133–139)	138 (136–141)	138 (134–140)	<0.001
**Albumin, ** ***g/dL***	3.0 (2.5–3.4)	2.8 (2.4–3.1)	3.8 (3.6–4.0)	5.3 (5.1–5.9)	<0.001
**Status at 3 months**					
Transplanted	24%	27%	15%	32%	<0.001
Died‡	11%	13%	5%	26%	<0.001

All data are median (IQR) or proportions (%).

* As reported by health care providers in hospitals where the patients were registered.

¥ Metabolic diseases include alpha-1-antitrypsin deficiency, Wilson disease, hereditary hemochromatosis, glycogen storage disorders, homozygous hypercholesterolemia, tyrosinemia, primary oxalosis, maple syrup urine disease, and other unspecificied metabolic disorders.

† Serum sodium available in 30,012 patients (74%).

‡ Kaplan-Meier mortality estimate with censoring at transplantation.

### Serum Albumin, MELD, and Mortality

The median follow-up period was 7.9 months (IQR 1.3–25.2) from the date of waiting list registration. In total, 3,690 patients (9%) died and 9,850 (24%) underwent liver transplantation within 90 days. Unadjusted Kaplan-Meier estimates of mortality at 3 months were 5% in patients with normal serum albumin, 13% in those with hypoalbuminemia, and 26% among hyperalbuminemic patients (*P*<0.0001). For serum albumin concentrations between 1.0 and 4.0 g/dL, an approximately linear, inverse relationship was observed between serum albumin and 3-month mortality after adjusting for MELD (Figure 2). Mortality increased significantly in patients with serum albumin concentrations above 4.0 g/dL. A similar relationship was observed after adjustment for MELDNa (Figure S1). With serum albumin bounded between 1.0 and 4.0 g/dL, the risk of death increased 44% per 1 g/dL decrease in serum albumin concentration after adjustment for MELD and other covariates (hazard ratio [HR] 1.44; 95% CI 1.35–1.54). This effect, which was significant when assessed at 1 year and during the entire follow-up period (data not shown), was greatest among patients with the lowest MELD scores due to an interaction between serum albumin and MELD (*β* = 0.038; *P*<0.0001).

### Model Performance

Based on Cox models for 90-day mortality, we derived the MELD-albumin and 5vMELD scores (see the Appendix for formulas). In the derivation cohort, the c-statistics of these models for predicting 90-day mortality were 0.910 (95% CI 0.901–0.919) and 0.922 (0.912–0.932), respectively. Table 2 includes c-statistics for the models for predicting mortality at 90 days, 1 year, and during the entire follow-up period in the validation cohort. For all outcomes, models including albumin (MELD-albumin and 5vMELD) outperformed their counterparts without albumin (MELD and MELDNa). The 5vMELD was the most discriminative model whereas MELD was least discriminative. The c-statistics for MELDNa and MELD-albumin were similar. Similar findings were noted in model comparisons within specific liver disease categories (Table S1).

**Table 2:** C-Statistics (95% CI) of MELD and Alternative Models for Predicting Mortality on the Liver Transplant Waiting List*.

Outcome	MELD	MELD-Albumin	MELDNa	5vMELD
**90-day mortality**	0.896 (0.884–0.908)	0.913 (0.903–0.923)†	0.912 (0.901–0.923)†	0.922 (0.912–0.931)† ^,^ ‡
**1-year mortality**	0.795 (0.783–0.807)	0.825 (0.814–0.835)†	0.821 (0.809–0.832)†	0.838 (0.828–0.849)† ^,^ ‡
**Overall mortality**	0.691 (0.681–0.701)	0.722 (0.712–0.731)†	0.716 (0.707–0.726)†	0.735 (0.725–0.744)† ^,^ ‡

* Analyses restricted to 15,114 patients from validation cohort with complete laboratory data.

† *P*<0.001 compared to MELD.

‡ *P*<0.001 compared to MELDNa.

### Reclassification of 90-Day Mortality Risk

Table 3 illustrates the risk reclassification of patients according to 5vMELD versus MELD, the current basis for organ allocation. Compared with MELD, NRI was 14.4% (95% CI 11.4 to 17.5%) for 5vMELD, 9.6% (95% CI 6.7 to 12.5%) for MELDNa, and 5.6% (95% CI 2.8 to 8.3%) for MELD-albumin (all *P*<0.0001 vs. MELD). As illustrated in Table 3, MELD categorized 51% of patients into the 0 to <5% risk stratum, 22% into the 5 to <10% stratum, 11% into the 10 to <20% stratum, and 17% into the ≥20% stratum. For 5vMELD, these figures were 58%, 14%, 10% and 18%, respectively. Overall, 3,921 patients (26%) were reclassified using 5vMELD instead of MELD (Table 3). Of these, 3,591 patients (92%) were considered correctly reclassified because observed mortality was closer to that predicted by 5vMELD than MELD. For example, 507 patients were ‘up-classified’ from the 5% to <10% mortality risk category using MELD to the 10% to <20% risk stratum using 5vMELD. Observed mortality in these patients (which made up 16% of this MELD stratum) was 11.0%, which falls into the 10% to <20% risk category. The average estimated risk for these patients with MELD was 7.7% compared with 13.0% using 5vMELD, which is closer to the 11.0% observed risk. As another example, 5vMELD ‘down-classified’ 1,550 patients from the 5% to <10% risk stratum using MELD to the 0% to <5% stratum. The mortality rate of 1.9% in these patients (who comprised 47% of this MELD group) falls into the 0% to <5% risk category. Again, the average estimated risk for these patients with 5vMELD (3.1%) was closer to observed mortality (1.9%) than that predicted with MELD (6.6%), suggesting beneficial reclassification with 5vMELD. In total, 15.6% of patients who died were correctly reclassified to a higher risk category using 5vMELD, whereas 8.4% were incorrectly reclassified to a lower risk category (classification improvement = 15.6%−8.4% = 7.2%). On the contrary, 16% of survivors were correctly down-classified whereas 8.8% were incorrectly up-classified (improvement = 16%−8.8% = 7.2%).

**Table 3:** Risk Reclassification Table Comparing 90-Day Mortality Risk Strata According to MELD and 5vMELD *.

	5vMELD				
MELD	0% to <5%	5% to <10%	10% to <20%	≥20%	Total
**0% to <5%**					
Persons included, *% (n)*	92.7% (7085)	5.9% (447)	1.5% (112)	0.04% (3)	50.6% (7647)
Deaths, *% (n)* *	73.4% (105)	16.1% (23)	10.5% (15)	0% (0)	12.9% (143)
Survivors, *% (n)* *	93.5% (6642)	5.5% (389)	1.0% (74)	0.01% (1)	65.8% (7106)
Observed mortality, *%* †	1.5%	5.4%	15.5%	0%	1.9%
**5% to <10%**					
Persons included, *% (n)*	47.0% (1550)	34.7% (1144)	15.4% (507)	3.0% (99)	21.8% (3300)
Deaths, *% (n)* *	18.8% (26)	36.2% (50)	33.3% (46)	11.6% (16)	12.4% (138)
Survivors, *% (n)* *	51.7% (1287)	34.1% (850)	12.6% (313)	1.6% (40)	23.1% (2490)
Observed mortality, *%* †	1.9%	5.0%	11.0%	22.1%	4.8%
**10% to <20%**					
Persons included, % (n)	4.9% (80)	27.7% (458)	42.1% (695)	25.3% (418)	10.9% (1651)
Deaths, % (n) *	2.7% (5)	14.8% (27)	42.1% (77)	40.4% (74)	16.4% (183)
Survivors, % (n) *	7.1% (60)	34.6% (294)	42.2% (359)	16.2% (138)	7.9% (851)
Observed mortality, % †	6.5%	7.1%	14.3%	26.9%	14.4%
**≥20%**					
Persons included, *% (n)*	0% (0)	1.0% (26)	8.9% (224)	90.1% (2266)	16.7% (2516)
Deaths, *% (n)* *	0% (0)	0.6% (4)	4.9% (32)	94.5% (613)	58.3% (649)
Survivors, *% (n)* *	0% (0)	3.1% (11)	23.7% (84)	73.2% (260)	3.3% (355)
Observed mortality, *%* †	0%	22.5%	21.0%	52.1%	47.8%
**Total**					
Persons included, *% (n)*	57.7% (8715)	13.7% (2075)	10.2% (1538)	18.4% (2786)	100% (15114)
Deaths, *% (n)*	12.2% (136)	9.3% (104)	15.3% (170)	63.2% (703)	100% (1113)
Survivors, *% (n)* *	74.0% (7989)	14.3% (1544)	7.7% (830)	4.1% (439)	100% (10802)
Observed mortality, *%* †	1.6%	5.7%	14.2%	45.2%	---

* Deaths and survivors at 90 days of follow-up, ignoring censored observations.

† Observed mortality at 90-days estimated from Kaplan-Meier curve using all observations within each cell. In total, 3921 patients (26%) were reclassified according to 5vMELD (in cells with ≥20 observations); 3591 (92%) were correctly reclassified. The reclassification calibration statistic for MELD is 273.2 (*P*<0.0001) vs. 60.0 for 5vMELD (*P*<0.0001). Reclassification improvement with 5vMELD is 7.2% (174−94 of 1113) among deaths and 7.2% (1736 – 955 of 10802) among survivors, leading to a net reclassification improvement of 14.4% (95% CI 11.4 to 17.5%; *P*<0.0001).

Table S2 includes a risk reclassification table of 5vMELD versus MELDNa. In total, 2,123 patients (14%) were reclassified using 5vMELD instead of MELDNa; 1,521 of these patients (72%) were correctly reclassified. NRI was 5.4% (95% CI 3.0 to 7.8%) for 5vMELD compared with MELDNa (*P*<0.0001).

Table 4 shows the distribution of scores among the 1,113 patients from the validation cohort who died within 3 months. In 37% (n = 414), the difference in scores was sufficient that their priority for transplantation may have increased had 5vMELD been in use. Based on the observed probabilities of transplantation in the validation cohort, an estimated 106 of these patients (26%) would have been transplanted, thus potentially preventing 9.5% (106/1,113) of these deaths. Had MELDNa been the basis for organ allocation instead of MELD, only an estimated 62 deaths (5.6%) of deaths would have been averted (Table S3).

**Table 4:** MELD and 5vMELD Scores among 1,113 Patients from the Validation Cohort Who Died on the Waiting List.

	5vMELD					
MELD	<10	10–19	20–29	30–39	40	Total
**<10**	6	24	5	0	0	**35**
**10–19**	0	62	216	3	0	**281**
**20–29**	0	0	226	166	0	**392**
**30–39**	0	0	0	253	0	**253**
**40**	0	0	0	0	152	**152**
**Total**	**6**	**86**	**447**	**422**	**152**	**1113**

During the study period, the probability of transplantation within 3 months of listing among patients in the validation cohort was 3.0% in patients with MELD<10, 11.4% with MELD 10–19, 46.9% with MELD 20–29, and 61.3% with MELD 30–39. If 5vMELD had been used to allocate donor organs instead of MELD, an estimated 106 additional transplantations would have been performed as calculated according to the following formula: 24×(11.4%−3.0%)+5×(46.9%−3.0%)+216×(46.9%−11.4%)+3×(61.3%−11.4%)+166×(61.3%−46.9%). Therefore, 9.5% of the deaths (106/1,113) that occurred with 3 months of listing might have been prevented had 5vMELD been used instead of MELD.

### Potential Survival Benefit of 5vMELD in Patients with Low MELD Scores

**Figure 3:**
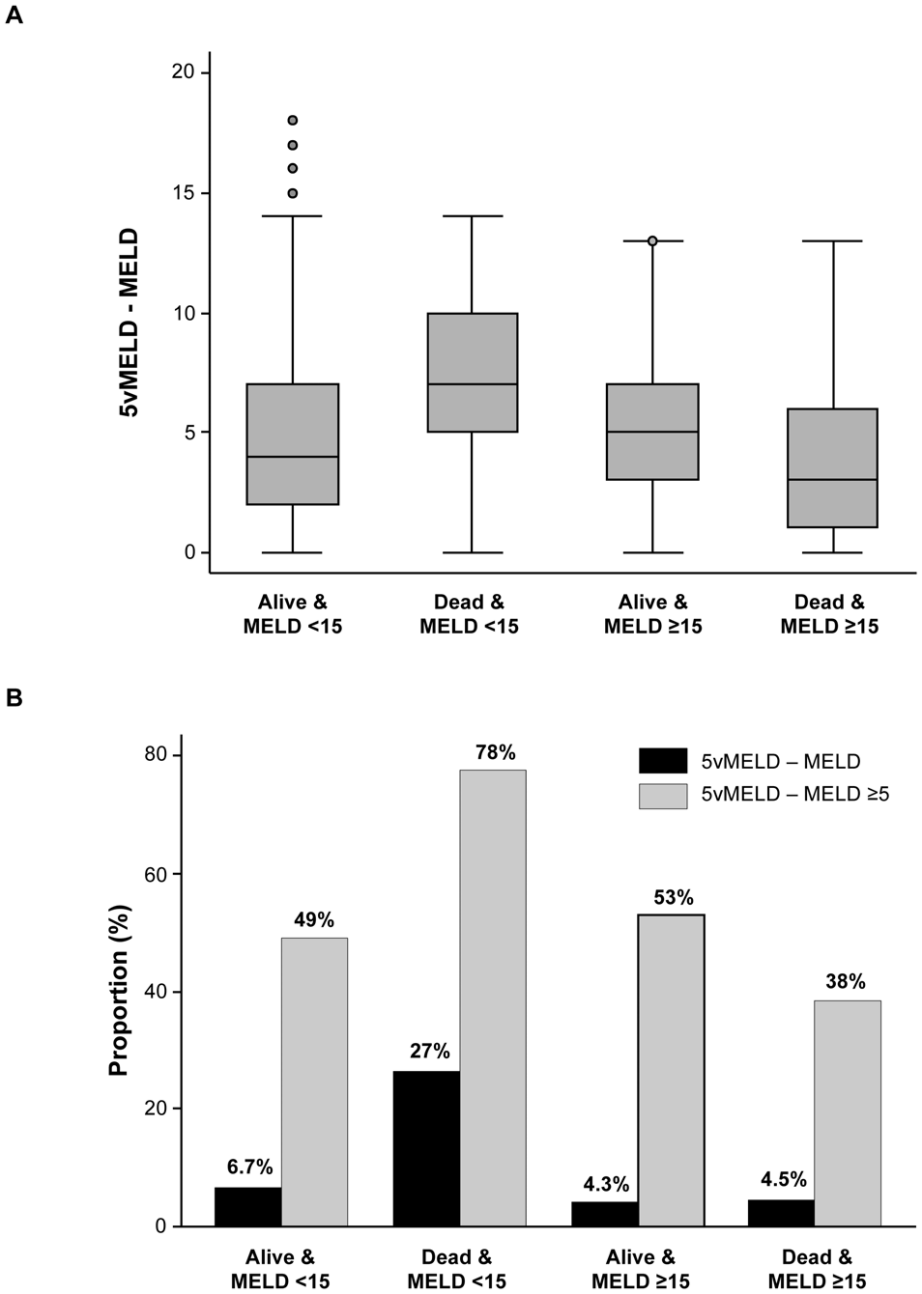
Difference between 5vMELD and MELD according to MELD score (< vs. ≥15) at waiting list registration and 3-month mortality. (A) Patients who died despite low MELD scores would have had the largest increase in points had a 5vMELD-based policy been in place (*P* = 0.0001). (B) The proportion of patients with a difference between 5vMELD and MELD of ≥10 and ≥5 points was highest among low MELD patients who died (both *P*<0.0005).

In total, 2% (143/7,647) of patients with MELD <15 died within 3 months of listing compared with 13% (970/7,467) with MELD ≥15 (*P*<0.0005) in the validation cohort. Compared with low MELD patients who survived, deceased patients had lower median serum albumin (2.8 vs. 3.3 g/dL) and sodium (136 vs. 138 mmol/L; both *P*<0.00005). Overall, the median difference between 5vMELD and MELD was 5 points (IQR 3–7). As demonstrated in Figure 3, patients who died despite low MELD scores would have had the largest increase in points had a 5vMELD-based allocation policy been in place (*P* = 0.0001). Among low MELD decedents, 27% would have gained ≥10 points under a 5vMELD-based system versus only 4.3% to 6.7% in the other groups (*P*<0.0005).

## Discussion

The current approach to liver transplantation in many countries is to allocate organs to those at the highest risk of death as predicted by the MELD score. Although the introduction of MELD has been associated with a significant decline in deaths on the waiting list [7], approximately 10% of registrants die within 3 months while they await an available organ. As such, a variety of modifications to MELD have been proposed in order to optimize the distribution of available organs [8], [9], [11], [12], [13], [14], [23]. Using a database of all potential liver transplant recipients in the United States, we describe the association between serum albumin, MELD and mortality among patients with end-stage liver disease. As expected, approximately three-quarters of patients were hypoalbuminemic and a low serum albumin concentration was an important predictor of mortality. Specifically, for every 1 g/dL reduction in the serum albumin concentration (between 1.0 and 4.0 g/dL), a 44% increase in the adjusted risk of 3-month mortality was observed (Figure 2). Due to an interaction between serum albumin and MELD, the impact of hypoalbuminemia fell as the MELD score increased. A similar interaction between MELD and serum sodium is the basis for MELDNa [13]. For example, in a patient with refractory ascites, a MELD score of 10, a serum sodium concentration of 125 mmol/L, and a serum albumin concentration of 2.0 g/dL, the MELDNa score would be 21 and 5vMELD score would be 26 (differences from MELD of 11 and 16 points, respectively). However, if this patient had a high MELD score of 30, the same sodium and albumin values would lead to only small increases in MELDNa to 33 and 5vMELD to 35.

In light of the independent association between hypoalbuminemia and waiting list mortality, we derived and validated novel prediction models including MELD and MELDNa with the serum albumin concentration. Based on their c-statistics, scores including albumin outperformed those without albumin (Table 2). 5vMELD was the most discriminative model (c-statistic for 90-day mortality, 0.922), while MELD had the lowest c-statistic (0.896). The benefit of adding albumin to MELD was similar to that of adding sodium to MELD (in MELDNa) and albumin to MELDNa (in 5vMELD). Based on these results, the use of 5vMELD to prioritize liver allocation instead of MELD or MELDNa may reduce mortality among patients on the waiting list. However, the potential benefit of such a shift in organ allocation policy is not completely evident in a comparison of c-statistics, which have been criticized for insensitivity and a lack of clinical relevance [21]. Strictly speaking, the c-statistic can be interpreted as the probability that the predicted risk for a randomly selected patient who dies is greater than that of a randomly selected survivor; the clinical importance of this distinction is limited [17], [18]. Therefore, we also constructed risk reclassification tables comparing our novel risk scores with MELD [19], [20], [21], [22]. This statistical approach to assessing risk prediction models has been adopted in many fields including cardiology [24], oncology [25], genetics [26], [27], and diabetes [28]. As demonstrated in Table 3, if 5vMELD had been used instead of MELD to prioritize patients for transplantation, 26% would have been reclassified. Reclassification in these patients would have been deemed correct in 92% of cases because their predicted risk of death with 5vMELD was closer to observed mortality than that estimated with MELD. Mirroring the c-statistic results, NRI compared with MELD was greatest using 5vMELD (14.4% vs. 9.6% with MELDNa). According to this analysis, 15.6% of patients who died were correctly reclassified to a higher risk category using 5vMELD, whereas 8.4% were incorrectly reclassified to a lower risk category. On the contrary, 16% of survivors were correctly down-classified whereas 8.8% were incorrectly up-classified. Based on calculations using crude scores rather than mortality risk predictions [13], we estimate that in the best-case scenario, approximately 10% of deaths (106/1,113) within 3 months of listing may have been prevented had 5vMELD been used instead of MELD (Table 4). This represents a 71% improvement over MELDNa, which may have prevented approximately 62 deaths (6%). The potential survival benefit of 5vMELD is greatest in patients with low MELD scores. Although these patients are traditionally deemed to have a low risk of death, they may have a significant mortality risk in settings with long waiting times. As demonstrated in Figure 3, 5vMELD is better able to identify these higher-risk, low MELD transplant candidates that would not otherwise be identified by MELD. Although we cannot ascertain the cause of death in these patients, we assume that hypoalbuminemia reflects the severity of liver disease plus other issues such as malnutrition, which will influence mortality in this patient population.

In addition to these important benefits for mortality prediction, the revision of MELD to include serum albumin is appealing for several reasons. First, hypoalbuminemia is an important marker of hepatic dysfunction, malnutrition, and the acute-phase response, which are common among patients with end-stage liver disease. Numerous studies have confirmed the prognostic importance of hypoalbuminemia among patients with [29], [30], [31], [32], [33], [34] and without [35] cirrhosis. Second, serum albumin is widely available and objective, and is measured using similar methods in nearly all U.S. laboratories (i.e. dye-binding with bromcresol green [BCG] or purple [BCP]). In general, these assays give very similar results and variability is low (within- and between-subject coefficients of variation <5%) [36], [37], [38]. For example, according to the College of American Pathologists 2010 Clinical Chemistry Surveys of 4,433 U.S. laboratories, the mean difference between the BCG and BCP methods for a standard of ∼3.0 g/dL (the median in our cohort) was only 0.1 g/dL and the coefficient of variation was 4.7% [39]. This variability is insufficient to significantly alter 5vMELD scores. However, since the BCP method may underestimate albumin in patients on hemodialysis [40] and with conjugated hyperbilirubinemia [41], the impact of different albumin assay methods and the inter-laboratory variability of 5vMELD should be investigated. Importantly, similar concerns have been raised regarding the components of MELD, particularly serum creatinine and INR, leading some to advocate standardization of these assays by UNOS [42], [43], [44].

A potential disadvantage of including serum albumin in liver allocation decisions is that intravenous albumin administration - a common practice in patients with end-stage liver disease - may lower a patient's likelihood of transplantation. Indeed, our finding of increased mortality and more severe hepatic and renal dysfunction among hyperalbuminemic patients likely reflects the administration of albumin to severely ill individuals. Importantly, this issue also applies to the survival benefit model for transplantation [34] and the CTP and PELD scores; yet PELD is used for organ allocation in children without reported disparities in transplant access due to albumin administration [6]. Moreover, like serum albumin, the other components of MELD and MELDNa may also be influenced by extraneous factors. For example, sodium and creatinine fluctuate widely due to changes in volume status and diuretic therapy, and the INR may rise due to warfarin or prolonged antibiotic treatment. Finally, albumin administration is most common in patients with the most severe liver disease including those with hepatorenal syndrome. In these patients - who tend to have high MELD and MELDNa – albumin infusions will have a minimal effect on 5vMELD due to the interaction term in its formula (see above). For example, in the patient described previously (sodium 125 mmol/L, albumin 2.0 g/dL, MELD 30, 5vMELD 35), a doubling of the serum albumin to 4.0 g/dL via intravenous infusion would yield only a 1-point reduction in 5vMELD. Nonetheless, an approach to mitigate this potential limitation would be to calculate 5vMELD using a patient's albumin value prior to any albumin administration (e.g. upon hospital admission among inpatients) or their nadir value (e.g. in outpatients with refractory ascites treated with large volume paracenteses), akin to assigning a creatinine of 4 g/dL in MELD for patients on dialysis. These approaches would obviate potential ‘gaming’ of the system – in this case, withholding intravenous albumin so as to not lower a patient's priority for transplantation. Importantly, regardless of this issue, 5vMELD still outperformed both MELD and MELDNa for the prediction of mortality throughout the follow-up period of our study.

Our study has several limitations. First, our findings depend on the validity of the data; an important consideration because the STAR database is a waiting list registry that was not designed specifically for this analysis. However, periodic audits at each center mandated by UNOS should ensure the validity of the data. Second, a significant number of patients were excluded due to missing laboratory tests, predominantly serum sodium concentrations. Patients with missing sodium were more likely to be listed during the initial part of the study period (2002–2004) and tended to have higher MELD scores and lower serum albumin concentrations, indicative of more severe disease. As a result, these patients had higher likelihoods of both death and transplantation at 3 months (data not shown). Therefore, the applicability of our results to these and other excluded subgroups (e.g. patients with acute liver failure, hepatocellular carcinoma, and exception cases) requires confirmation. Third, due to the extensive validation and widespread familiarity of MELD among the transplant community, we developed our new models including albumin without refitting the coefficients of the original scores. Nevertheless, models with new coefficients for these variables did not improve discrimination over 5vMELD (data not shown). Similarly, we derived our novel models based on laboratory results at waiting list registration rather than as time-dependent covariates. We chose this approach to remain consistent with the existing literature and to avoid deriving overly complex models that would not be useful in routine practice. Future studies should consider fluctuations in these scores throughout the period on the waiting list when comparing their predictive utility. Finally, the benefits of adopting 5vMELD over initiatives unrelated to risk stratification (e.g. expanded organ sharing) could not be evaluated in our study.

In summary, our data confirms the important negative prognostic impact of hypoalbuminemia among liver transplant candidates after adjusting for the MELD score and serum sodium concentration. Compared with MELD, novel risk scores including serum albumin, particularly 5vMELD, improve the prediction of short-term mortality among patients awaiting liver transplantation. If validated and shown to be associated with reduced mortality on the transplant waiting list, adoption of 5vMELD as the basis for liver allograft allocation may improve outcomes on the liver transplant waiting list.

## Supporting Information

Figure S1 **Serum albumin concentration and the risk of death within 3 months of waiting list registration after adjustment for the MELDNa score. Dotted lines represent 95% confidence intervals.**
(TIFF)

Table S1 **C-statistics (95% CI) of MELD and alternative models for predicting 3-month mortality on the liver transplant waiting list according to liver disease etiology.**
(DOC)

Table S2 **Risk reclassification table comparing 90-day mortality risk strata according to MELDNa and 5vMELD.**
(DOC)

Table S3 **MELD and MELDNa scores among 1,113 patients from the validation cohort who died on the waiting list.**
(DOC)

Appendix S1 **Formulas for MELD, MELDNa, MELD-Albumin, and 5vMELD.**
(DOC)
