# Supplementary figures and images for: Revision of MELD to Include Serum Albumin Improves Prediction of Mortality on the Liver Transplant Waiting List
Source: PLoS One. 2013 Jan 18;8(1):e51926. doi: 10.1371/journal.pone.0051926 (PMC3548898; doi:10.1371/journal.pone.0051926)

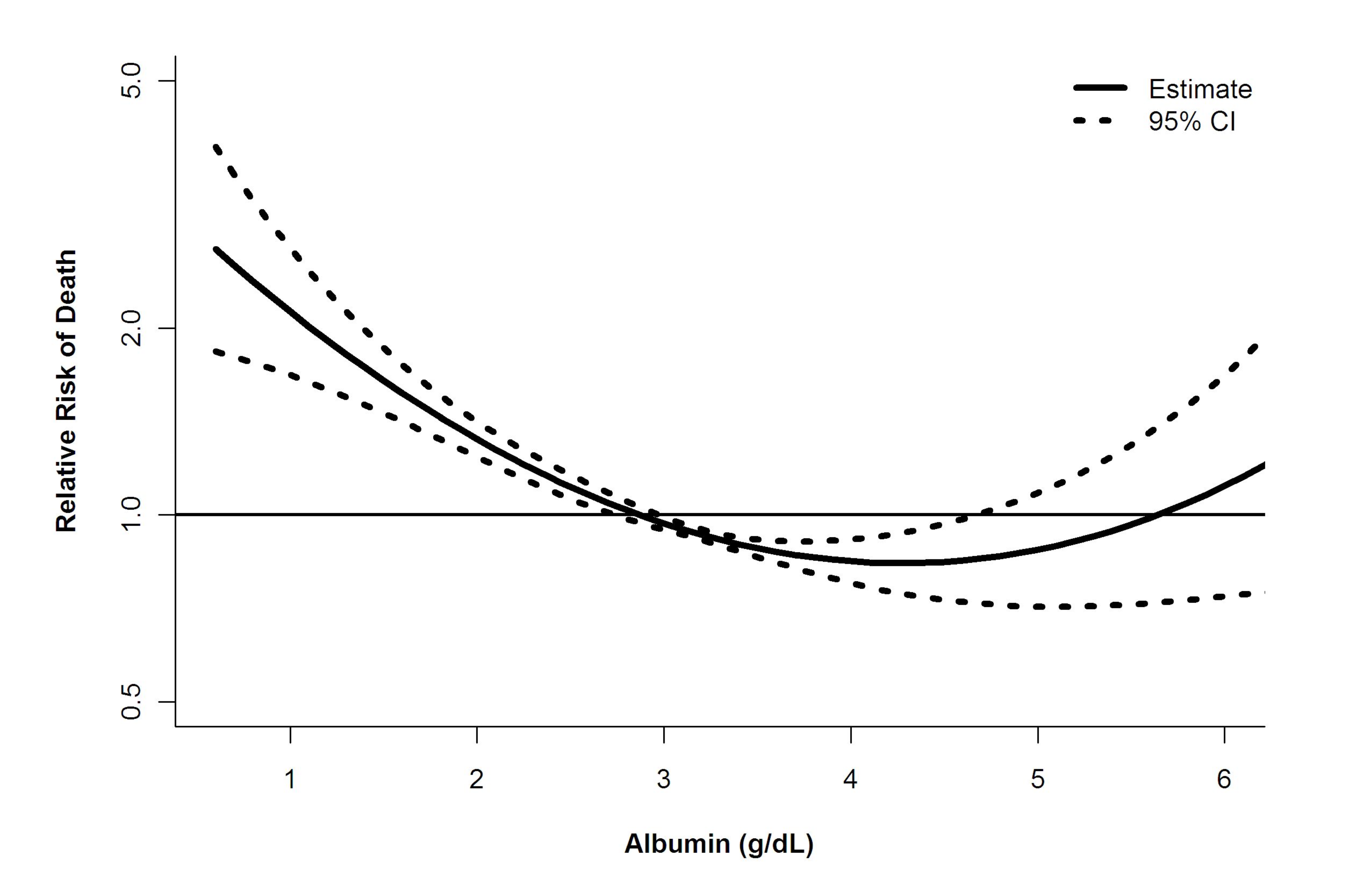

Supplement: Figure S1 — Serum albumin concentration and the risk of death within 3 months of waiting list registration after adjustment for the MELDNa score. Dotted lines represent 95% confidence intervals. (TIFF) [file pone.0051926.s001.tiff]
