# Supplementary material for: Revision of MELD to Include Serum Albumin Improves Prediction of Mortality on the Liver Transplant Waiting List
Source: PLoS One. 2013 Jan 18;8(1):e51926. doi: 10.1371/journal.pone.0051926 (PMC3548898; doi:10.1371/journal.pone.0051926)
Supplement: Table S1 — C-statistics (95% CI) of MELD and alternative models for predicting 3-month mortality on the liver transplant waiting list according to liver disease etiology. (DOC) [file pone.0051926.s002.doc]

Table S1: C-Statistics (95% CI) of MELD and Alternative Models for Predicting 3-Month Mortality on the Liver Transplant Waiting List According to Liver Disease Etiology *

| **Outcome** | **MELD** | **MELD-Albumin** | **MELDNa** | **5vMELD** |
| --- | --- | --- | --- | --- |
| **Hepatitis C (n=6,159)** | 0.902 (0.883-0.920) | 0.912 (0.896-0.928) ^†^ | 0.916 (0.899-0.933) ^†^ | 0.922 (0.906-0.938) ^†,‡^ |
| **Alcohol (n=2,552)** | 0.907 (0.880-0.934) | 0.920 (0.896-0.943) | 0.922 (0.898-0.945) | 0.930 (0.909-0.951) ^†^ |
| **Cholestatic (n=1,361)** | 0.927 (0.894-0.959) | 0.930 (0.894-0.966) | 0.938 (0.909-0.967) ^†^ | 0.937 (0.905-0.969) |
| **Other (n=5,042)** | 0.885 (0.864-0.905) | 0.909 (0.893-0.925) ^†^ | 0.903 (0.885-0.921) ^†^ | 0.916 (0.901-0.932) ^†,‡^ |

* Analyses restricted to 15,114 patients from the validation cohort with complete laboratory data.

^†^ *P*<0.05 compared to MELD.

^‡^ *P*<0.05 compared to MELDNa.
