# Supplementary material for: Revision of MELD to Include Serum Albumin Improves Prediction of Mortality on the Liver Transplant Waiting List
Source: PLoS One. 2013 Jan 18;8(1):e51926. doi: 10.1371/journal.pone.0051926 (PMC3548898; doi:10.1371/journal.pone.0051926)
Supplement: Table S2 — Risk reclassification table comparing 90-day mortality risk strata according to MELDNa and 5vMELD. (DOC) [file pone.0051926.s003.doc]

**Table S2: Risk Reclassification Table Comparing 90-Day Mortality Risk Strata According to MELDNa and 5vMELD ***

|  | **5vMELD** | | | |  |
| --- | --- | --- | --- | --- | --- |
| **MELDNa** | **0% to <5%** | **5% to <10%** | **10% to <20%** | **≥20%** | **Total** |
| **0% to <5%** |  |  |  |  |  |
| Persons included, *% (n)* | 93.1% (8343) | 6.7% (602) | 0.2% (15) | 0% (0) | 59.3% (8960) |
| Deaths, *% (n)* * | 82.1% (128) | 16.0% (25) | 1.9% (3) | 0% (0) | 14.0% (156) |
| Survivors, *% (n)* * | 93.8% (7684) | 6.0% (493) | 0.1% (11) | 0% (0) | 75.8% (8188) |
| Observed mortality, *%* ^†^ | 1.6% | 4.6% | 19.9% | 0% | 1.8% |
| **5% to <10%** |  |  |  |  |  |
| Persons included, *% (n)* | 15.6% (372) | 56.8% (1357) | 27.1% (648) | 0.5% (11) | 15.8% (2388) |
| Deaths, *% (n)* * | 5.6% (8) | 49.7% (71) | 44.1% (63) | 0.7% (1) | 12.9% (143) |
| Survivors, *% (n)* * | 18.4% (305) | 58.9% (979) | 22.6% (375) | 0.2% (3) | 15.4% (1662) |
| Observed mortality, *%* ^†^ | 2.3% | 6.0% | 12.0% | 22.1% | 7.0% |
| **10% to <20%** |  |  |  |  |  |
| Persons included, *% (n)* | 0% (0) | 9.6% (116) | 65.5% (790) | 24.9% (300) | 8.0% (1206) |
| Deaths, *% (n)* * | 0% (0) | 5.1% (8) | 60.3% (94) | 34.6% (54) | 14.0% (156) |
| Survivors, *% (n)* * | 0% (0) | 12.2% (72) | 69.3% (408) | 18.5% (109) | 5.5% (589) |
| Observed mortality, *%* ^†^ | 0% | 8.6% | 15.5% | 26.3% | 17.2% |
| **≥20%** |  |  |  |  |  |
| Persons included, *% (n)* | 0% (0) | 0% (0) | 3.3% (85) | 96.7% (2475) | 16.9% (2560) |
| Deaths, *% (n)* * | 0% (0) | 0% (0) | 1.5% (10) | 98.5% (648) | 59.1% (658) |
| Survivors, *% (n*) * | 0% (0) | 0% (0) | 9.9% (36) | 90.1% (327) | 3.4% (363) |
| Observed mortality, *%* ^†^ | 0% | 0% | 17.8% | 48.5% | 47.2% |
| **Total** |  |  |  |  |  |
| Persons included, *% (n)* | 57.7% (8715) | 13.7% (2075) | 10.2% (1538) | 18.4% (2786) | 100% (15114) |
| Deaths, *% (n)* | 12.2% (136) | 9.3% (104) | 15.3% (170) | 63.2% (703) | 100% (1113) |
| Survivors, *% (n)* * | 74.0% (7989) | 14.3% (1544) | 7.7% (830) | 4.1% (439) | 100% (10802) |
| Observed mortality, *%* ^†^ | 1.6% | 5.7% | 14.2% | 45.2% | --- |

* Deaths and survivors at 90 days of follow-up, ignoring censored observations.

^†^ Observed mortality at 90-days estimated from Kaplan-Meier curve using all observations within each cell. In total, 2123 patients (14%) were reclassified according to 5vMELD (in cells with ≥20 observations); 1521 (72%) were correctly reclassified. The reclassification calibration statistic for MELDNa is 93.1 (*P*<0.0001) vs. 52.6 for 5vMELD (*P*<0.0001). Reclassification improvement with 5vMELD is 10.8% (146 - 26 of 1113) among deaths and -5.4% (413 – 991 of 10802) among survivors, leading to a net reclassification improvement of 5.4% (95% CI 3.0 to 7.8%; *P*<0.0001).
