# Supplementary material for: Revision of MELD to Include Serum Albumin Improves Prediction of Mortality on the Liver Transplant Waiting List
Source: PLoS One. 2013 Jan 18;8(1):e51926. doi: 10.1371/journal.pone.0051926 (PMC3548898; doi:10.1371/journal.pone.0051926)
Supplement: Table S3 — MELD and MELDNa scores among 1,113 patients from the validation cohort who died on the waiting list. (DOC) [file pone.0051926.s004.doc]

**Table S3: MELD and MELDNa Scores among 1,113 Patients from the Validation Cohort Who Died on the Waiting List**

|  | **MELDNa** | | | | |  |
| --- | --- | --- | --- | --- | --- | --- |
| **MELD** | **<10** | **10-19** | **20-29** | **30-39** | **40** | **Total** |
| **<10** | 14 | 21 | 0 | 0 | 0 | **35** |
| **10-19** | 0 | 142 | 139 | 0 | 0 | **281** |
| **20-29** | 0 | 0 | 313 | 79 | 0 | **392** |
| **30-39** | 0 | 0 | 0 | 253 | 0 | **253** |
| **40** | 0 | 0 | 0 | 0 | 152 | **152** |
| **Total** | **14** | **163** | **452** | **332** | **152** | **1113** |

During the study period, the probability of transplantation within 3 months of listing among patients in the validation cohort was 3.0% in patients with MELD<10, 11.4% with MELD 10-19, 46.9% with MELD 20-29, and 61.3% with MELD 30-39. If MELDNa had been used to allocate donor organs instead of MELD, an estimated 62 additional transplantations would have been performed as calculated according to the following formula: 21 x (11.4%-3.0%) + 139 x (46.9%-11.4%) + 79 x (61.3%-46.9%). Therefore, 5.6% of the deaths (62/1,113) that occurred with 3 months of listing might have been prevented had MELDNa been used instead of MELD.
