# Supplementary material for: Revision of MELD to Include Serum Albumin Improves Prediction of Mortality on the Liver Transplant Waiting List
Source: PLoS One. 2013 Jan 18;8(1):e51926. doi: 10.1371/journal.pone.0051926 (PMC3548898; doi:10.1371/journal.pone.0051926)
Supplement: Appendix S1 — Formulas for MELD, MELDNa, MELD-Albumin, and 5vMELD. (DOC) [file pone.0051926.s005.doc]

**APPENDIX: Formulas for MELD, MELDNa, MELD-Albumin, and 5vMELD**

**MELD** = 11.2 x ln(INR) + 3.78 x ln(bilirubin, *in mg/dL*) + 9.57 x ln(creatinine, *in mg/dL*) + 6.43

Lower limit of 1.0 for all variables and creatinine capped at 4.0 mg/dL (including for patients on renal replacement therapy).

**MELDNa** = MELD – Na – (0.025 x MELD x [140-Na]) + 140

Same rules as MELD for bounding of variables and serum sodium bounded between 125 and 140 mmol/L.

**MELD-Albumin** = MELD + (6.179 x [4-albumin]) – (0.163 x MELD x [4-albumin])

Albumin is bounded between 1 and 4 g/dL.

**5vMELD** = MELDNa + (5.275 x [4-albumin]) – (0.136 x MELDNa x [4-albumin])

Albumin is bounded between 1 and 4 g/dL.
